# Potential of Peroxisome Proliferator-Activated Receptor Gamma Antagonist Compounds as Therapeutic Agents for a Wide Range of Cancer Types

**DOI:** 10.1155/2008/494161

**Published:** 2008-09-02

**Authors:** Jack D. Burton, David M. Goldenberg, Rosalyn D. Blumenthal

**Affiliations:** ^1^Coney Island Hospital, 2601 Ocean Parkway, Brooklyn, NY 11235, USA; ^2^Center for Molecular Medicine and Immunology, 520 Belleville Avenue, Belleville, NJ 07109, USA

## Abstract

PPAR*γ* is a therapeutic target that has been exploited for treatment of type II diabetes mellitus (T2DM) with agonist drugs. Since PPAR*γ* is expressed by many hematopoietic, mesodermal and epithelial cancers, agonist drugs were tested and shown to have both preclinical and clinical anticancer activities. While preclinical activity has been observed in many cancer types, clinical activity has been observed only in pilot and phase II trials in liposarcoma and prostate cancer. Most studies address agonist compounds, with substantially fewer reports on anticancer effects of PPAR*γ* antagonists. In cancer model systems, some effects of PPAR*γ* agonists were not inhibited by PPAR*γ* antagonists, suggesting noncanonical or PPAR*γ*-independent mechanisms. In addition, PPAR*γ* antagonists, such as T0070907 and GW9662, have exhibited antiproliferative effects on a broad range of hematopoietic and epithelial cell lines, usually with greater potency than agonists. Also, additive antiproliferative effects of combinations of agonist plus antagonist drugs were observed. Finally, there are preclinical in vivo data showing that antagonist compounds can be administered safely, with favorable metabolic effects as well as antitumor effects. Since PPAR*γ* antagonists represent a new drug class that holds promise as a broadly applicable therapeutic approach for cancer treatment, it is the subject of this review.

## 1. INTRODUCTION

PPAR*γ* is one of the three known peroxisome proliferator-activated receptors and is a member of the nuclear receptor (NR) superfamily. Since it has a predominantly nuclear location, regardless of whether cognate ligands are present, it is classified as a type II NR. It functions as a transcription factor by heterodimerizing with the retinoid X receptor (RXR), after which this complex binds to specific DNA sequence elements called peroxisome proliferator response elements (PPREs) [1]. In order to become fully active as a transcription factor, PPAR*γ* must be bound by ligand. RXR can be affected by binding its own cognate ligands, usually resulting in incremental increases in transcriptional activity. After the PPAR*γ*/RXR heterodimer binds to PPREs in promoter regions of target genes, coactivator proteins, such as p300 (CBP), SRC-1, and Drip205 (or TRAP220) family members, are recruited to this complex to modulate gene transcription [2–4]. Different PPAR*γ* ligands appear to be able to recruit different coactivators, which may explain differences in the biological activity between ligands [5].

The cardinal biologic activity of PPAR*γ* is the induction of differentiation of adipocytes, the cell type that expresses the highest levels of PPAR*γ* amongst normal tissues. Lower levels of PPAR*γ* are, however, found in other normal tissues and cell types such as skeletal muscle, liver, breast, prostate, colon, type 2 alveolar pneumocytes, some endothelial cells as well as monocytes, and B-lymphocytes. There are three *PPAR*γ** mRNA isoforms (*γ*1, *γ*2, and *γ*3) and two major protein species (*γ*1 and *γ*2). The mRNA isoforms are generated by alternate promoter usage, resulting in an additional 28 amino acids at the N-terminus of PPAR*γ*2 compared with PPAR*γ*1. Most tissues express PPAR*γ*1, whereas the PPAR*γ*2 isoform is expressed mostly by adipocytes. The longer N-terminal domain of PPAR*γ*2 may affect function, since this isoform was shown to confer a higher level of ligand-independent transcriptional activity, which was further increased by physiologic concentrations of insulin [6]. High levels of PPAR*γ* expression by fat and its role in adipogenesis led to the recognition that agonistic PPAR*γ* ligands have antidiabetic effects. The chemical class of PPAR*γ* agonists known as thiazolidinediones (TZDs) demonstrated high-affinity binding to PPAR*γ* [7] as well as favorable therapeutic properties, and such drugs were eventually registered for the treatment of type II diabetes mellitus (T2DM). Three TZD drugs have been registered in the U.S.: rosiglitazone (Avandia), pioglitazone (Actos), and troglitazone(Rezulin). Subsequent to its marketing and widespread use, troglitazone was associated with idiosyncratic and, in rare cases, fatal hepatic toxicity, and, thus, was withdrawn from the market. The former two drugs, however, have remained as safe and effective therapeutic options for the management of T2DM.

Not long after reports of the cloning of PPAR*γ* and its expression in normal tissues [8, 9], PPAR*γ* expression was observed in an array of primary cancers and derivative cell lines. Its expression was reported initially in liposarcoma [10], and soon thereafter in colon, breast, and prostate carcinomas and additional cancer types [11–14]. In addition to the in vitro and preclinical in vivo anticancer effects of TZDs, pilot clinical studies using troglitazone showed antitumor activity in patients with liposarcoma and prostate cancer [15, 16]. Compounds from other chemical classes were also shown to bind PPAR*γ* and to have antiproliferative effects in cancer models, such as the naturally occurring eicosanoid, 15-deoxy-Δ^12,14^-prostaglandin J_2_(15-d-PGJ_2_), the N-aryl tyrosine derivative, GW1929 [17], and the triterpenoid, 2-cyano-3,12-dioxooleana-1,9-diene-28-oic acid, CDDO [18]. While compounds that exhibit PPAR*γ* agonist activity, such as TZDs, have PPAR*γ*-dependent antiproliferative effects, they have also been shown to have antiproliferative effects in cell types that are genetically PPAR*γ*-null [19]. Also, uncertainty about mechanisms of anticancer effects of PPAR*γ* ligands has resulted from variability in the classification of some compounds (e.g., bisphenol A diglycidyl ether [BADGE], which has been shown to have both agonist and antagonist activities) [20, 21].

## 2. EFFECTS OF PPAR*γ* ANTAGONIST COMPOUNDS IN EPITHELIAL CANCER MODEL SYSTEMS: CELL GROWTH AND APOPTOSIS

The initial report of Fehlberg et al. [22] showed an inhibitory effect of this class of agents on a colon cancer and a lymphoma cell line using the compound, BADGE, which as noted has been classified as both an agonist and antagonist. This initial study did not examine effects on proliferation, but showed that apoptotic effects, such as increases in annexin-V binding and reductions in DNA content as assessed by propidium iodide staining, required 50–100 *μ*M concentrations of BADGE, which would tend to increase off-target effects. Subsequently, Seargent et al. [23] showed that a higher affinity, selective PPAR*γ* antagonist, GW9662, had direct antiproliferative effects on three breast cancer cell lines of differing phenotypes (ER+, ER−, and p53-null). This antagonist compound was somewhat more potent in its effects than an agonist (rosiglitazone). In this report, the role of PPAR*γ* in mediating growth inhibition was addressed, but not fully elucidated. All three cell lines expressed it and the predicted, canonical PPAR*γ*-related transactivation effects were demonstrated, with the agonist inducing transactivation and the antagonist suppressing it, thus excluding PPAR*γ*-mediated transactivation as the mechanism of this effect. There are data, however, that suggest that antagonist-type compounds may also act via other PPAR*γ*-dependent pathways. Lea et al. reported similar results using a range of agonist and antagonist compounds on both murine and human cell lines [24]. Schaefer et al. showed that the antiproliferative effect of the PPAR*γ* antagonist, T0070907, on hepatocellular carcinoma cell lines was attenuated by knockdown of PPAR*γ* by siRNA [25]. These data are consistent with a PPAR*γ*-mediated transrepression mechanism, which has been demonstrated with respect to anti-inflammatory effects of PPAR*γ* ligands mediated by the NF-*κ*B signaling pathway. Pascual et al. showed similar effects of a pure agonist (rosiglitazone) and a mixed agonist/antagonist (GW0072) on the repression of a NF-*κ*B-regulated gene, *iNOS*, suggesting that pure antagonists may also be capable of mediating this effect [26].

There are also data that PPAR*γ* ligands (both agonist and antagonist) exert PPAR*γ*-independent effects suggesting other cellular targets of these compounds. This was demonstrated clearly by Palakurthi et al., who demonstrated in vitro and in vivo growth inhibition of two agonist compounds, troglitazone and ciglitazone, in experiments utilizing PPAR*γ* ^−/−^ and PPAR*γ* ^+/+^ embryonic stem cell lines (ES), both of which exhibited very similar sensitivity to these compounds [19]. This effect was shown to be mediated in part by the inhibition of the initiation of protein translation, since these TZD compounds increased the phosphorylation and consequent inactivation of elongation-initiation factor 2 (eIF2) both in cells that expressed and were null for PPAR*γ*. The effect of antagonist compounds on this pathway has not been reported. As noted, BADGE had similar proapoptotic effects in a colon cancer line expressing PPAR*γ* and a T-lymphoma line that showed no detectable expression of it (by immunoblotting and RT-PCR) of this target [22]. But, given the variable classification of this compound as both an antagonist and agonist, the mechanism underlying this effect and its attribution are unclear.

## 3. OTHER EFFECTS OF PPAR*γ* ANTAGONIST COMPOUNDS

PPAR*γ* antagonist compounds have also been shown to affect cell shape, adhesion, and invasiveness of cancer cell lines. Masuda et al. evaluated the effects of the PPAR*γ* antagonists, BADGE, GW9662 and T0070907, on four squamous carcinoma cell lines derived from tumors of the oral cavity. Antiproliferative effects were shown for the three antagonists, but not for the agonist, pioglitazone [27]. Effects of these agents on adhesion and anoikis were also evaluated. Antagonists were found to inhibit adhesion and induce cell death related to loss of adhesion (known as anoikis) under normal tissue culture conditions on untreated plastic dishes. T0070907 induced similar inhibition of adhesion to fibronectin-coated plates, and this was significantly reversed by coincubation of cells with this antagonist and the agonist, pioglitazone, suggesting a PPAR*γ*-dependent effect. Since adhesion and detachment are related to cytoskeletal structure and function, this was assessed by fluorescent staining of F-actin. Using confocal microscopy, T0070907 was shown to cause dose-dependent disruption of F-actin, associated with rounding of the cells. Additional experiments showed inhibition of FAK and MEK-ERK signaling pathways, as well as decreased expression of integrin *α*5 and CD151, both of which are adhesion proteins that have been implicated in cancer cell invasion and metastasis. Schaefer et al. showed similar effects of PPAR*γ* antagonists on hepatocellular carcinoma cell lines including inhibition of adhesion, induction of anoikis, and inhibition of phosphorylation and activation of FAK [25]. These effects were shown to be dependent on the degree of PPAR*γ* inhibition, and could be mediated by the antagonist or knockdown of PPAR*γ* via specific, cognate siRNA. T0070907 was also shown to have substantially greater growth inhibitory effects on the HepG2 line compared with the agonist drugs, troglitazone, and rosiglitazone. Takahashi et al. demonstrated anti-invasive and growth inhibitory effects of the antagonists, GW9662 and T0070907, on esophageal cancer cell lines. The anti-invasive effects were observed at levels substantially lower than those required for growth inhibition [28]. In summary, all of these studies addressing anticancer effects of PPAR*γ* antagonist compounds have show effects on cell growth, adhesion, and invasion in multiple epithelial cancer models.

Some of these effects are PPAR*γ*-dependent, but the potential role of other targets is suggested by the similar effects of BADGE on a PPAR*γ*+ colon cancer line and a PPAR*γ*-negative T-lymphoma line. Also, the substantially different concentrations of PPAR*γ* antagonists needed to induce anti-invasive effects versus growth inhibition in esophageal cancer lines suggest different mechanisms with differing degrees of PPAR*γ* dependence or lack of involvement of the PPAR*γ*-signaling pathway for some effects. A PPAR*γ*-independent effect of antagonists on colorectal cancer cell lines and in an in vivo tumor xenograft derived from one of the lines was shown in a more recent report by Schaefer et al. [29]. A decrease in tubulin levels was observed that was independent of PPAR*γ*, PPAR*δ*, and proteasome function. This downregulation of tubulins *α* and *β* may explain the antimigratory, anti-invasive, and antimetastatic effects that were observed. Thus, in summary, PPAR*γ* antagonist compounds with varying chemical structures (though GW9662 and T0070907 are similar) have several significant anticancer effects in vitro and in vivo in epithelial cancer model systems including breast, colon, aerodigestive squamous cell, and hepatocellular.

## 4. EFFECTS OF PPAR*γ* ANTAGONISTS IN HEMATOPOIETIC CANCER MODEL SYSTEMS

Studies were conducted in our lab to assess the effects of PPAR*γ* antagonists on hematopoietic cell lines. Initial screening showed that several myeloma (MM) cell lines had the greatest sensitivity to the antiproliferative effects of the antagonists, GW9662 and T0070907. Thus multiple MM lines were tested, including one that is IL-6-dependent, for sensitivity to these compounds as well as to the agonist, pioglitazone. MM lines as well as non-Hodgkin lymphoma (NHL) lines showed significantly greater sensitivity to the growth inhibitory effects of the two antagonist drugs compared with the agonist [30]. As a group, the MM lines were more sensitive than the other groups of cancer cell lines to the antiproliferative effects of the antagonists, particularly T0070907. Other goals were to directly compare the sensitivity of previously tested epithelial cancer types (breast and colon) to hematopoietic lines (MM and NHL) as well as to evaluate a chemoresistant epithelial cancer type (renal cell). These experiments showed that in all the epithelial and hematopoietic cell lines tested, the antagonists were significantly more potent in their growth inhibitory effects compared with the agonist drug.

The IC_50_ values for the panel of 16 cell lines tested in these studies are shown in Table 1. For each of the cell lines in the panel, significant differences in the IC_50_ values of the antagonist compounds and the agonist drug, pioglitazone, were observed (*P* values ranging from <.04 to <.001, with 12 of 16 lines at <.001). While the MM lines showed the greatest sensitivity to the antagonists, similar degrees of sensitivity to the antagonists were also seen in the subset of breast cancer lines, which included two lines that are estrogen receptor-negative. Though not quite as sensitive as a subset, significant differences between the antagonists and the agonist were also observed in the renal cell lines, which are among the most chemoresistant epithelial lines. The differential sensitivities within and across cell lines did not appear to be related to the levels of PPAR*γ* expression. Also, neither the agonist nor the antagonist induced significant upregulation of PPAR*γ* as has been reported in some studies with PPAR*γ* ligands. Consistent with prior reports, combinations of the agonist and with each of the antagonists did not result in attenuation of growth inhibitory effects. In fact, schedule-dependent increases in growth inhibition were observed, particularly when the antagonists were added to cells 24 hours prior to the agonist. Aspects of the mechanisms of cytotoxicity of the antagonists and agonists were also compared. It was shown that both classes of PPAR*γ* ligand-induced apoptotic effects, but this effect was found to be caspase-independent for the agonist, pioglitazone [30].

Another question that was addressed was the impact of IL-6 on the responses of the MM lines to PPAR*γ* antagonists, since this is a cytokine that plays a central role in the pathogenesis and progression of MM, as well as other cancer types. For these studies, 4 of the 5 MM lines that were utilized were IL-6-independent in order to follow up on a previous report of Wang et al. that analyzed the responses of three MM lines to the PPAR*γ* agonists, 15-d-PGJ_2_ and troglitazone. This report showed that growth inhibition and certain downstream signaling events were PPAR*γ*-dependent, and also that two IL-6-dependent MM lines expressed PPAR*γ* while an IL-6-independent line did not [31]. Also, GW9662 was reported to block the effects of the agonists, and had no antiproliferative activity on its own. We utilized five different MM lines, of which four are IL-6 independent (CAG, KMS12-BM, KMS12-PE, and OPM-6) as well as a fifth that is dependent on an IL-6 autocrine loop (U266B1). In contrast to the prior report cited above, of the lines analyzed, CAG expressed PPAR*γ*, while the autocrine IL-6-dependent line, U266B1, did not express PPAR*γ* by immunoblotting. Also, three of the four of IL-6-independent MM lines were more sensitive to the growth inhibitory effects of both of the two PPAR*γ* antagonist compounds compared with the IL-6-dependent line, U266B1 (see Table 1).

In MM cell lines, which are more often IL-6 dependent compared with other B cell lines, the strict dependence on exogenous IL-6 is indicative of ongoing requirement for this signaling pathway, which in pathophysiologic states, such as MM, usually depends on production of this cytokine by stromal cells. In MM, clinically more aggressive or treatment-resistant disease is associated with production of IL-6 by the myeloma cell themselves as opposed to the bone marrow stroma [32]. MM lines show a spectrum of IL-6 dependence, with some being dependent on exogenous IL-6, others being dependent on its autocrine production, and yet others being IL-6-independent for their growth. Even those MM lines that are not strictly dependent on IL-6 for their growth (exogenous or autocrine) can still be affected by the addition of exogenous IL-6 [33] (also shown in one of the lines tested, OPM-6, [34]). Addition of IL-6 to such MM lines has been shown to induce either incremental stimulation of proliferation or induction of resistance to various agents such as dexamethasone, standard chemotherapy drugs such as melphalan and other agents. Thus the interaction of IL-6 and PPAR*γ* antagonist compounds were examined in two MM lines (KMS12-PE and OPM-6). MTT assays were performed in the presence and absence of exogenous IL-6 (5 ng/mL). For both of these MM lines, addition of IL-6 did not induce resistance, but instead appeared to increase the sensitivity of these lines to T0070907, with a similar trend observed with GW9662 [30].

## 5. DOSE-RESPONSE EFFECTS OF PPAR*γ* ANTAGONIST COMPOUNDS AND INTERACTION WITH OTHER AGENTS

The PPAR*γ* antagonist compounds, GW9662 and T0070907, differ in their antiproliferative dose-response effects compared with the agonist as well as other agents. Not only are the corresponding IC_50_ values for the antagonists significantly lower than the agonist, pioglitazone, but a greater degree of growth inhibition (85–97% versus 50–80%) was observed with the former compounds. Also, of note was that the maximal effects of these agents were seen at concentrations that were only 2- to 3-fold greater than the IC_50_ across the entire panel of cell lines tested that included cell lines with relative and very high levels of chemoresistance (colon and renal cell, resp.). The dose-response curves were much steeper with the antagonist compounds compared to the agonist, pioglitazone, and also much steeper than what is observed with most other agents, including standard chemotherapy drugs and other agents (see Figure 2). This dose-response relationship suggests either a positive cooperative effect, potentially via increased, cooperative recruitment of corepresssors, thereby increasing transrepression. The alternate possibility is that different targets are being engaged with gradually increasing concentrations, which together exhibit additive or supra-additive interactions.

Since MM lines as a group were the most sensitive of the cell lines we tested, interaction with other novel agents for therapy of MM were evaluated. One such agent is anti-CD74 monoclonal antibody (mAb). CD74 was shown to be strongly expressed by the malignant plasma cells in the vast majority of clinical MM specimens as well as the majority of MM lines [35]. It was also shown that this mAb in unlabeled (cold) form exhibited in vitro growth inhibitory effects on both NHL and MM lines [36]. The anti-CD74 mAb used in these studies, LL1, also showed significant therapeutic effects in two preclinical murine NHL xenograft models. In preliminary in vitro studies, the humanized anti-CD74 mAb was combined with T0070907 in two MM lines. These studies also evaluated a sixth MM line (KMS11), which is IL-6 independent, expresses CD74 and is useful as a murine MM xenograft model. This line showed similar sensitivity to T0070907 as the other IL-6-independent lines, with an (unpublished observations, J Burton). Another IL-6-independent MM line that was used in initial studies, KMS12-PE, was also used to evaluate interactions between T0070907 and the hLL1 mAb. While KMS11 line showed moderate sensitivity to hLL1 (maximum growth inhibition of 50–70%), the KMS12-PE line was resistant to single-agent hLL1 (<10% inhibition). However, in combination with T0070907, there was a sizable shift to the left of the dose-response curve, as is shown in one representative experiment in Figure 2. Current data indicate that the IC_50_ value decreases by from a mean value of ~4.1 *μ*M for T0070907 alone versus ~3.0 *μ*M with T0070907 in combination with hLL1, suggestive of a supra-additive effect (25–30% observed versus <8% expected based effect of hLL1 alone). This is a promising initial preclinical lead given that hLL1 is now being evaluated in several phase I/II clinical trials in B-cell cancers such as NHL and MM, and appears to be safe and well tolerated.

## 6. OVERVIEWOF MECHANISMS OF ACTION OF PPAR*γ* AGONIST AND ANTAGONIST COMPOUNDS

The studies reviewed above have shown that the effects of PPAR*γ* ligands are mediated by various mechanisms. Some studies show or suggest canonical PPAR*γ*-mediated effects (i.e., via transactivation), as exemplified by early in vitro studies with agonist compounds that showed fat accumulation, a major PPAR*γ*-mediated effect, in both breast cancer and liposarcoma cell lines [10, 12]. This was also demonstrated in liposarcoma patients in whom increased fat content within tumors was demonstrated by serial CT scanning before and after treatment with an agonist drug [10]. The studies of Wang et al. showed that the growth-inhibitory effects of PPAR*γ* agonist compounds on MM lines was seen only in lines expressing PPAR*γ* and that these effects were reversed by cotreatment with an antagonist compound [31]. In contrast, completely PPAR*γ*-independent effects were demonstrated for both agonist and antagonist compounds in reports from Palakurthi et al. [19] and Schaefer et al. [29]. This was clearly shown for the agonist compounds, troglitazone and ciglitazone, which showed similar antiproliferative effects in PPAR*γ*-wild type and PPAR*γ*-null (knockout) embryonic stem cell lines, both in vitro and in vivo [19]. PPAR*γ*-independent growth inhibitory and antimetastatic effects of several antagonist compounds were shown in both in vitro and in vivo studies using three colon carcinoma cell lines. These effects were associated with reductions in tubulin levels and were also shown to be independent of PPAR*δ* and proteasome function. The PPAR*γ*-independent effect of agonist compounds was shown to be associated with inhibitory effects on the protein translation pathway. The mechanism of PPAR*γ*-independent effects of antagonist compounds on tubulin levels has not been elucidated.

The mechanism of PPAR*γ*-mediated transrepression may explain some of the effects of antagonist compounds. This was suggested by the attenuation of the effects of antagonist compounds by PPAR*γ* knockdown by siRNA in hepatocellular carcinoma cell lines [25]. Also, the observation that combinations of PPAR*γ* agonist and antagonist compounds result in additive antiproliferative effects in various cancer cell lines [24, 30] is consistent with this mechanism. This mechanism is plausible, as it has been shown to inhibit the NF-*κ*B signaling pathway, which is central to inflammation and to the proliferation and survival of multiple cancer types including hepatocellular and colon carcinomas as well as multiple myeloma. The potential role of this and other mechanisms remain to be determined.

## 7. SUMMARY OF PRECLINICAL STUDIES OF PPAR*γ* ANTAGONIST COMPOUNDS AND THEIR CLINICAL POTENTIAL

The studies reviewed above have shown that PPAR*γ* antagonists have in vitro and preclinical in vivo anticancer effects that are as broad and potent as agonist compounds. These effects have been demonstrated in a wide range of epithelial cancer cell lines as well as hematopoietic cancer cell lines. Exploration of the underlying mechanisms of action for antagonist compounds has shown either involvement of PPAR*γ* or a PPAR*γ*-independent effect. One study suggested the involvement of the canonical transactivation mechanism in that antagonist effects were antagonized by coincubation with an agonist compound, pioglitazone [27]. In another study, where knockdown of PPAR*γ* affected responses to antagonist compounds, the effect was not consistent with the canonical transactivation mechanism, but may be consistent with a transrepressive mechanism [25]. Another study showed that anticancer effects were associated with reductions in tubulin levels (a validated cancer-related target), but this was not mediated by PPAR*γ*, PPAR*δ*, or the proteasome [29].

**Figure 1:**
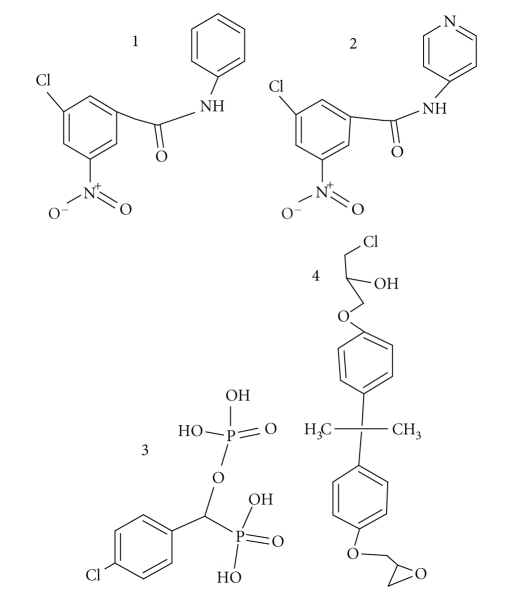
The chemical structures of four PPAR*γ* antagonists: (1) GW9662, (2) T0070907, (3) SR-202, and (4) BADGE.

**Figure 2:**
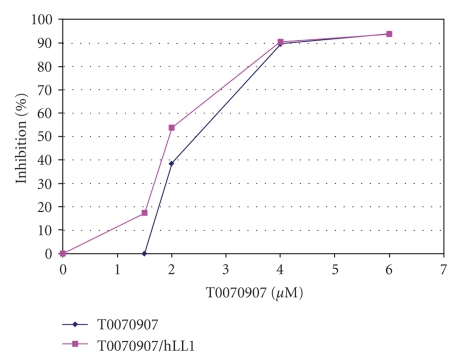
Dose-response curves for the MM line, KMS12-PE, to T0070907, both in the presence and absence of the hLL1 mAb. Square symbols represent the dose-response curve in the presence of hLL1, and diamond symbols represent the curve in the absence of LL1. The ordinate shows percent growth inhibition values and the abscissa the concentration of T0070907 in micromolar.

While there have been numerous preclinical in vivo studies in cancer models with PPAR*γ* agonists, there have been relatively few with antagonist compounds. Also agonists have been tested clinically. Some studies with antagonists have been conducted in noncancer models at low doses (≤1 mg/kg), which were not toxic and biologically active [37, 38]. A chemically distinct, but selective PPAR*γ* antagonist, SR-202, has been synthesized and evaluated in preclinical models (Figure 1). It was given at a dose of 400 mg/kg for periods of up to 10 weeks with favorable metabolic effects such protection against diet-induced hyperinsulinemia and reduction in hyperinsulinemia and hyperglycemia in genetically predisposed (ob/ob) mice [39]. In pilot studies, we have administered moderate doses of GW9662 (15 mg/kg) and T0070907 (7.5 mg/kg) daily for 3 weeks by the intraperitoneal route to immunodeficient mice. These doses and schedules were well tolerated and resulted in no signs of toxicity (unpublished observations). These data indicate that the doses of these antagonists that may be sufficient for anticancer therapy are well tolerated, paving the way for further development of these agents for treatment of cancer.

## Figures and Tables

**Table 1:** Mean IC_50_ values (*μ*M) for the PPAR*γ* ligands.

Cell lines	Pioglitazone	T0070907	GW9662
Colon			

Moser^#^	26.5 ± 2.6	15.9 ± 1.0	20.1 ± 0.3
HT29^§^	53.0 ± 4.7	11.2 ± 0.0	14.1 ± 0.5
LS174T^#^	38.7 ± 7.4	7.8 ± 1.9	9.5 ± 0.5
HCT-15^§^	53.1 ± 2.5	13.0 ± 0.5	19.0 ± 0.8

RCC			

A498^#^	38.9 ± 4.9	24.3 ± 0.7	29.1 ± 0.3
ClearCa-2^§^	56.4 ± 3.1	20.8 ± 1.9	21.5 ± 0.7

Breast			

ZR75-30^§^	77.9 ± 7.0	3.9 ± 0.3	10.6 ± 0.9
MCF7^§^	54.8 ± 3.9	10.2 ± 1.9	16.6 ± 2.4
MDA-MB-231^§^	78.7 ± 3.5	20.1 ± 1.1	26.8 ± 1.0

MM			

CAG*	62.4 ± 9.9	12.2 ± 1.2	13.8 ± 0.1
KMS12-BM^§^	33.2 ± 5.1	3.2 ± 0.6	11.8 ± 1.6
KMS12-PE^§^	56.4 ± 1.5	4.3 ± 0.3	9.5 ± 0.9
OPM6^§^	48.9 ± 1.8	4.1 ± 0.3	11.5 ± 0.1
U266B1^§^	56.6 ± 1.3	9.9 ± 0.2	29.7 ± 1.5

NHL			

Ramos^§^	66.5 ± 7.4	12.7 ± 0.7	15.1 ± 0.1
SU-DHL6^§^	53.1 ± 1.4	11.8 ± 0.4	14.8 ± 0.3

Mean IC_50_ values from replicate experiments with this panel of cells for each of the three PPAR*γ* ligands are shown above, expressed in *μ*M ± SEM. Cell lines are grouped according to cancer type. IC_50_ values from each cell line were compared by single factor ANOVA analysis, with all lines showing significant differences as indicated: ^§^ *P* < .0001; *P < .005; ^*#*^ *P* < .04.
